# The Action of Chemical Denaturants: From Globular to Intrinsically Disordered Proteins

**DOI:** 10.3390/biology12050754

**Published:** 2023-05-22

**Authors:** Antonella Paladino, Luigi Vitagliano, Giuseppe Graziano

**Affiliations:** 1 Institute of Biostructures and Bioimaging, CNR, Via Pietro Castellino 111, 80131 Naples, Italy; antonella.paladino@cnr.it (A.P.); luigi.vitagliano@cnr.it (L.V.); 2 Department of Science and Technology, University of Sannio, via Francesco de Sanctis snc, 82100 Benevento, Italy

**Keywords:** denaturants, urea, guanidinium, solvent, conformational ensemble, intrinsically disordered proteins

## Abstract

**Simple Summary:**

The ability of chemical denaturants to perturb protein structures is well-established, but the physico-chemical basis underlying this phenomenon is still debated. In the present review, we survey classical and recent literature to provide a global overview of the effects that chemical denaturants produce on the different structural states of proteins, from globular to intrinsically disordered and amyloid-like assemblies. Interestingly, the different forces that stabilize these distinct structural states generate intriguing effects. Even the ranking of the relative strength of the most common denaturants (i.e., urea and guanidinium ion), which is well-established and generally conserved for globular proteins, is not fully suited for other structural states. Analysis of available data, using both polymer physics and atomic-interaction-based perspectives, provides complementary and somehow convergent views of the mechanism of action of chemical denaturants. The different “quality” of water as a solvent in distinct contexts, and the remarkable promiscuity of chemical denaturants represent useful conceptual frameworks to shed light on these intricate phenomena.

**Abstract:**

Proteins perform their many functions by adopting either a minimal number of strictly similar conformations, the native state, or a vast ensemble of highly flexible conformations. In both cases, their structural features are highly influenced by the chemical environment. Even though a plethora of experimental studies have demonstrated the impact of chemical denaturants on protein structure, the molecular mechanism underlying their action is still debated. In the present review, after a brief recapitulation of the main experimental data on protein denaturants, we survey both classical and more recent interpretations of the molecular basis of their action. In particular, we highlight the differences and similarities of the impact that denaturants have on different structural classes of proteins, i.e., globular, intrinsically disordered (IDP), and amyloid-like assemblies. Particular attention has been given to the IDPs, as recent studies are unraveling their fundamental importance in many physiological processes. The role that computation techniques are expected to play in the near future is illustrated.

## 1. Introduction

Proteins combine a formidable molecular complexity with fine structural/functional regulation. Although they are typically composed of thousands of atoms, even the replacement of very few atoms may cause dramatic effects on their structural stability and/or their functionality. The many biological roles that these molecular giants perform in living organisms are generally interpreted through the “structure-function” paradigm which assumes that protein activity depends on the spatial localization of its atoms. The traditional picture of protein structures oscillating between a very limited number of functional folded structures (i.e., the native state) and an incredibly large number of unfolded conformations (i.e., the denatured state ensemble) has been progressively complicated by the discovery that these macromolecules frequently assume other, somewhat extreme, states [1,2,3].

A plethora of studies in the last two decades has indicated that a significant portion of proteins, especially among those encoded by the genomes of higher organisms, are endowed with such remarkable intrinsic flexibility that even the identification of well-defined 3D structural states becomes difficult [4,5]. These proteins, which populate a large number of conformations that rapidly interconverts one into the other, are commonly classified as intrinsically disordered proteins (IDPs) [1,2,6]. Almost simultaneously, careful structural investigations have shown the broad propensity of proteins to aggregate and assume extremely rigid states based on a structural motif denoted as cross-β [3]. Although the formation of these structures is connected to the misfolding process associated with widespread and severe neurodegenerative diseases, there is growing evidence of their functional relevance [7]. Therefore, depending on amino acid sequences and environmental conditions, proteins may exhibit distinct states characterized by a continuum of intrinsic flexibility and thermodynamic stability.

In this intricate scenario, characterized by a multitude of radically different manifestations of protein 3D structures, even the description of conformational stability, which should necessarily go beyond the folding/unfolding paradigm, requires novel conceptual frameworks. How the knowledge accumulated in decades of studies focused on the thermodynamics of globular proteins, which typically display an equilibrium between a minimal number of discrete (folded) states and a vast ensemble of high flexible (unfolded) conformations, can be translated either to hyper-rigid cross-β states or to highly flexible IDPs represents an intriguing open issue. Similarly, how this multitude of distinct structural states is affected by external modulators, such as chemical denaturants, is a topic of great interest and intense research activities [8,9,10,11].

**Figure 1:**
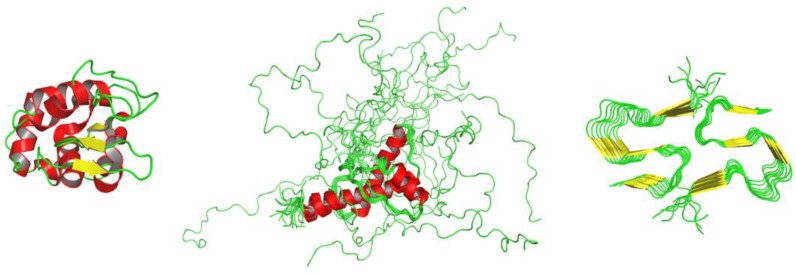
Different classes of protein structural states. From left to right side, a globular protein, an IDP ensemble, and an amyloid-like assembly are shown: lysozyme (PDB entry: 5i4y), Nuclear Magnetic Resonance (NMR) structure of the *wild-type* human prion protein (ensemble ID: PED00045, cross reference PDB entry: 5yj5 [12,13]), and Aβ40 (PDB entry: 5kk3). Available online: https://www.rcsb.org/, https://proteinensemble.org/ and last accessed on 20 April 2023.

In the present review, we survey classical and recent literature reports to provide a global overview of the effects that chemical denaturants produce on the different states populated by proteins (see Figure 1).

Indeed, the evaluation of the impact of denaturants on proteins exhibiting rather different structural behaviors may represent a valuable tool for the definition or the validation of the mechanism(s) underlying their action. Attention will be devoted to the differences and similarities in the action of chemical denaturants, specifically urea, and guanidinium, towards these radically distinct structural states. The choice is largely dictated by the availability of experimental, theoretical, and computational studies of these two denaturing agents on all the structural states populated by the polypeptide chains. Clearly, the number of published articles on these topics is incredibly large, and so a selection has been performed, mainly based on our expertise and knowledge, and personal preferences. Nevertheless, we hope to have been able to offer an interesting overview of such a large scenario, providing structural and physico-chemical interpretations of available experimental data.

## 2. Molecular Mechanism(s) of Chemical Denaturation of Globular Proteins

It is firmly established that the native, folded state of globular proteins is marginally more stable than the unfolded, denatured state in terms of Gibbs free energy [14,15,16,17] (i.e., for a 100-residue protein, the denaturation Gibbs free energy amounts to 40–60 kJ mol^−1^, at room temperature, corresponding to the energy of 2–3 H-bonds, adopting the value originally estimated by Pauling [18]). Relevant data comes from the possibility of reversibly destroying the native state, through both physical agents (i.e., temperature and pressure) and chemical agents. The denatured state is important for the protein conformational stability as much as the native state; since it cannot simply be described as a random coil, its structural features need to be clarified to arrive at a more complete understanding of folding, unfolding and misfolding [19]. Among the chemical denaturing agents, urea and guanidinium chloride (GdmCl) are the most used in research and industrial labs, for a long time. Nevertheless, there is still debate on the microscopic mechanism of their denaturing action. In this respect, some points need to be clearly assessed.

First, it is necessary to recognize that both urea and GdmCl are weak denaturing agents because, at room temperature, the full denaturation of a generic and stable globular protein with a concentration of about 10^−4^ M requires 2–6 M urea or GdmCl aqueous solutions. The difference of four orders of magnitude in concentration is impressive, even considering how large the surface of a protein is. Such a comparison does not imply the existence of a simple relationship between the protein concentration and the denaturant concentration required for denaturation. Rather, it points out the subtleties of the protein-denaturant interactions. Second, it is important to underscore that concentrated aqueous solutions of urea and GdmCl are very different from water. The addition of both urea or GdmCl to water causes a significant density increase [20,21], and a significant decrease in water molar concentration (as reported in Table 1). For instance, at 25 °C and 1 atm, a 6 M urea aqueous solution has d = 1088 g L^−1^ and [H_2_O] = 40.4 M, while a 6 M GdmCl aqueous solution has d = 1140 g L^−1^ and [H_2_O] = 31.5 M, to be contrasted with the values of pure water: d = 997 g L^−1^ and [H_2_O] = 55.3 M. The density increase implies that there are good attractive interactions between the water and urea on one hand and between water, guanidinium, and chloride ions on the other. Indeed, both urea and GdmCl are very soluble in water, and their addition causes an increase in surface tension compared to pure water (see Table 1) [22,23]. Moreover, since the first hydration shell of urea [24], Gdm^+^, and Cl^-^ ions [25] consists of about six water molecules, it should be clear that, in all 6 M aqueous solutions, most of the water molecules are involved in the hydration shell of these denaturants. Third, the above sentences and numbers should help to understand why there is still debate on the molecular mechanism of the denaturing action of urea and GdmCl.

However, the density increase caused by the addition of urea or GdmCl to water (coupled with the increase in volume packing density, the fraction of liquid volume really occupied by solvent and cosolvent molecules or ions) leads to an increase in the magnitude of the solvent-excluded volume effect that stabilizes the native state (i.e., the conformations possessing the smallest solvent-accessible-surface-area are stabilized for entropic reasons) [26,27]. Therefore, the denaturing action of both urea and GdmCl cannot come from changes in the solvent medium, but from direct interactions with protein surfaces. The occurrence of such attractive direct interactions emerged to rationalize the experimental solubility data of small molecules [28,29], and to construct reliable force-fields for both urea and GdmCl [30,31,32,33]. Recently, direct interactions established by these denaturants have been confirmed in several protein structures deposited in the Protein Data Bank (PDB) [34,35,36].

**Figure 2:**
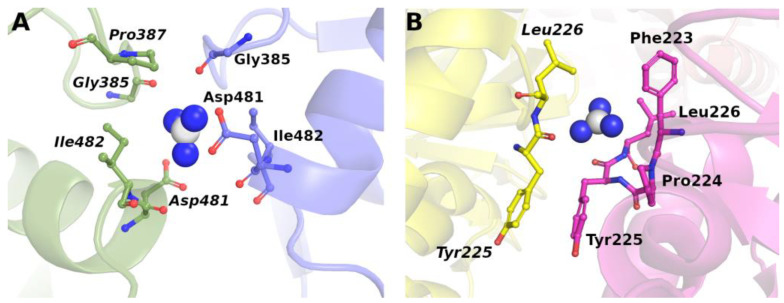
Example of guanidinium binding mode. Representative examples of protein structures where (**A**) Gdm^+^ establishes a large number of different interactions at the interface between the two chains in the Herpes Simplex DNA polymerase (PDB entry: 2gv9): H-bonds with CO of G385, two salt-bridges with Od1/Od2 of D481 and four hydrophobic contacts with P387, I482, G385; (**B**) Gdm^+^ is mainly stabilized by nonpolar contacts in Oxidoreductase (PDB entry: 6uw2), accessed on 15 February 2023. Gdm^+^ is displayed in white (carbon) and blue (nitrogen) spheres, interacting amino acids are given in ball-and-sticks while protein chains are rendered in cartoons.

Both urea molecules and Gdm^+^ ions have a planar geometry, with π electrons delocalized over the whole structure, and the ability to form several H-bonds. Urea, in particular, resembles a water dimer because it can be involved in six H-bonds, four acting as hydrogen donors and two acting as hydrogen acceptors [24]. On the other hand, the large polarizability, due to the presence of π electrons, allows the establishment of good van der Waals-type attractive interactions. Indeed, the analysis of the binding sites detected on the surface of the native protein structures indicates the promiscuous nature of both urea and Gdm^+^ ions, which can make H-bonds and van der Waals-type attractions with almost all the protein chemical moieties (see Figure 2 for representative examples of Gdm^+^ binding pockets).

**Figure 3:**
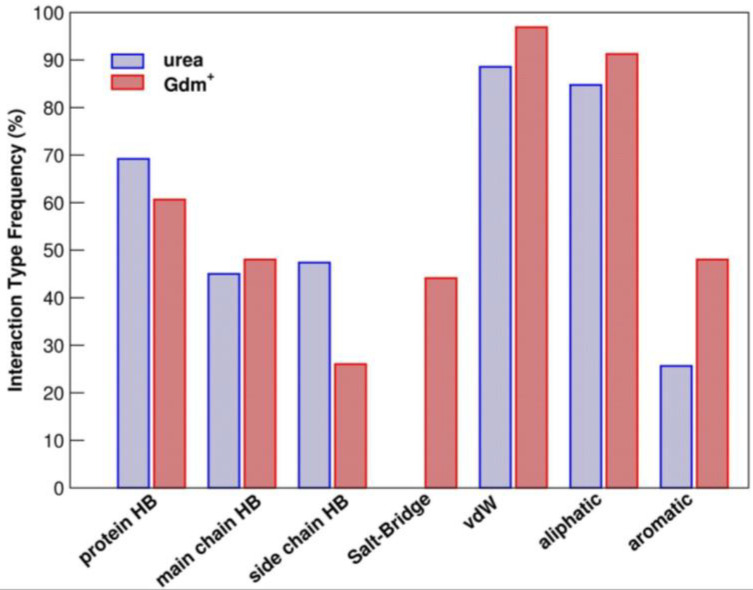
Frequency of interaction types established by urea and Gdm^+^ on protein surfaces. On the x-axis hydrogen bonds (HB) and van der Waal interactions (vdW) are distinguished based on the atom involved in the contact: protein main or side chain and aliphatic or aromatic moiety of the amino acid. Data are reported from previous PDB mining research [36] and are given as the percentage of analyzed ligand binding sites (i.e., 289 for urea, and 127 for Gdm^+^).

**Figure 4:**
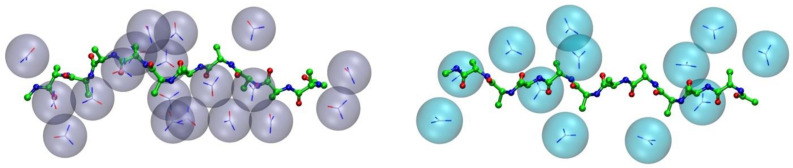
Urea and guanidinium in the hydration shell of a model polyalanine. Snapshots from all-atoms Molecular Dynamics (MD) simulations performed in 2 M urea (**left**) and 2 M GdmCl (**right**) aqueous solutions of a restrained 10mer polyalanine (Paladino et al., unpublished data). Stretches of alanine decapeptide were built imposing a down puckered conformation (ɸ~−75°, Ψ~145°) and kept in the extended shape with backbone atoms restrained by a harmonic potential (force constant = 1000 kJ/mol·nm^2^). MD simulations were performed using Gromacs package 2021 [44], amber99SB-ildn [45] force field, and TIP4P water model [46]. Simulation boxes include ~2600 water molecules and ~ 100 Urea/GdmCl molecules.

The PDB survey indicates that, on average, a urea molecule makes five contacts with protein groups, while a Gdm^+^ ion makes six contacts with protein groups, with a remarkable affinity for aromatic side chains (see Figure 2B, Figure 3 and Figure 4) [36]. This promiscuity is the ground of their denaturing action because the large surface exposed on unfolding is by no means different from that of the native state and so it markedly increases the number of available binding sites (note that promiscuity can also explain the action of the thiocyanate ion, a strong protein denaturant [35]). It is important to underscore that the average binding constant *per* site, for both urea molecules and Gdm^+^ ions, is small, only slightly larger than one, because the denaturants have to compete with water and replace some of the water molecules covering, with a dense monolayer, all the protein surface [37,38,39,40,41,42]. The analysis performed by Schellman on five globular proteins, at room temperature, led to the following average values: K_b_ = 1.2 M^−1^ for urea and 1.4 M^−1^ for Gdm^+^ ion [39]. Record and co-workers developed a solute partitioning model, distinguishing a local domain close to the protein surface from the bulk solution, to account for the coupling between denaturant binding and the exchange of water molecules; at room temperature, the average dimensionless partition coefficient, measuring the local-bulk concentration ratio, was around 1.2 for urea, and around 2.0 for Gdm^+^ ion [41,42,43]. Therefore, the average binding constant *per* site is larger for the Gdm^+^ ions, in line with their stronger denaturing action. The different strength should be a consequence of the tighter attractive interactions that urea makes with water; Gdm^+^ ions are characterized by a low charge density and so their attractive interactions with water are good, but not as good as those of urea [24,25,29,30,31,32,33]. This analysis implies that the driving force of denaturant binding to protein surfaces (and so of denaturation) is not due to energetic factors, but comes from the entropy gain due to the large number of configurational microstates created by the occupation and non-occupation of the binding sites [36,37]. It proves to be a direct but subtle mechanism that provides a reason why it has been so difficult to clarify.

Of course, somewhat different views of urea and Gdm^+^ denaturing action are present in the literature. For instance, Thirumalai and co-workers performed several studies to clarify the microscopic mechanism of the denaturing action of urea and GdmCl [47,48,49,50], by developing a simplified coarse-grained model of polypeptide chains in which each residue is represented by the alpha-carbon and a side chain carbon [51]. In the computational approach, there is no explicit water, whose effect is accounted for by a suitable parameterization that, by performing Langevin MD trajectories at different temperatures, calculating the canonical partition function and then the heat capacity, leads to values of the denaturation temperature close to the experimental ones (i.e., those recorded in DSC measurements). The action of the two denaturants is modeled using the experimental Gibbs free energy changes (associated with the transfer of backbone and side chains from water), to the desired urea or GdmCl aqueous solution (using literature data) and accounting for their interactions with the very large number of conformations sampled during the Langevin MD trajectories, whose accessibility depends on their water accessible surface area. In this respect, it is necessary to recognize that the transfer of Gibbs free energy changes are macroscopic thermodynamic quantities that cannot provide a microscopic mechanism. The calculated heat capacity peaks are similar to those of the DSC measurements and, on increasing the urea or GdmCl concentration, the values of denaturation temperature decrease, in line with the experimental data. Therefore, Thirumalai and co-workers claimed that the molecular transfer model, originally proposed by Tanford [52], works well in rationalizing the effect of urea and GdmCl. However, the height (and so the area) of the calculated heat capacity peaks increases by increasing the denaturant concentration [49,50], at odds with the experimental data. This failure should be considered an indication that something is not entirely correct in the computational-theoretical approach. Although the neglect of explicit water molecules, and of explicit urea molecules or Gdm^+^ and Cl^-^ ions in the MD simulations can allow a satisfactory sampling of the conformational space accessible to the simplified polypeptide models, the good computational performances do not necessarily guarantee a correct account of the physico-chemical mechanisms governing the conformational stability of polypeptide chains. These observations suggest that: (a) the subtleties of the fundamental hydrophobic effect cannot be fully replaced with a simple list of effective interaction parameters; (b) molecular interactions that proteins establish with solvent and denaturants are crucial for their conformational stability.

## 3. Denatured and Native States in the Polymer Physics Perspective

Polypeptide chains populating the denatured state do not have a single structure and, given the huge number of available conformations in the dihedral angle space, they have to be described as a statistical ensemble. There are large structural differences among the conformations belonging to the denatured state ensemble, and, for their description, it is necessary to use average quantities, i.e., the average radius of gyration, <Rg>. Thus, polymer physics could be useful to characterize both experimentally and theoretically the denatured state ensemble. Specifically, by resorting to the classic Flory’s mean-field treatment of homo-polymers [53,54], <Rg> should scale with the number of monomer units raised to an exponent whose value depends on the “quality” of the solvent in which the polymer is dissolved, <Rg> ∝ *N*^ν^, where *N* is the number of monomers in the chain. Quality is a word that should provide a measure of the strength of the monomer-monomer attractions in comparison to the strength of the monomer-solvent attractions. If the former is stronger, the polymer chains collapse to maximize the monomer-monomer contacts, the exponent ν = 1/3, and the solvent is classified as “poor”. If the monomer-solvent attractions are stronger, the polymer chains swell to maximize the contacts with the solvent, the exponent ν = 3/5, and the solvent is classified as “good”. If there is a perfect balance between the intramolecular and intermolecular attractions, the polymer chains do not swell nor collapse, the exponent ν = 1/2, and the solvent is termed a “theta” solvent. In the latter case, the chains behave as ideal chains of non-interacting segments and follow the Gaussian statistics of a simple 3D random walk. Actually, it is necessary to account for the volume occupied by each monomer unit and the fact that a given spatial position cannot be occupied by two different monomer units at the same time means that an intra-chain excluded volume effect is operative and strongly reduces the number of available conformations; thus, the correct model to describe chain conformations is a 3D self-avoiding random walk [55]. By including the entropic excluded volume effect in the Gibbs free energy balance governing the polymer energetics, the above-reported dependences between the average radius of gyration and the number of monomer units continue to be valid.

Such polymer physics ideas have also been applied to describe the native state and the denatured state of the foldable proteins, and also the ensembles populated by IDPs (see below). A word of caution is necessary. It should be clear that proteins are hetero-polymers, usually dissolved in water, and the latter is a special solvent because it can make strong H-bonds with peptide groups and several side chains, and entropically favor compact conformations to reduce the solvent-excluded volume effect caused by the simple presence of a chain molecule in the liquid [56,57,58]. Nevertheless, by analyzing the 3D folded structures deposited in the PDB, Dima & Thirumalai and Holehouse & Pappu found that <Rg(N-state)> ≈ 3(Å)·*N*_res_^1/3^ [59,60], as expected for chains in a poor solvent.

Moreover, small-angle X-ray scattering (SAXS), small-angle neutron scattering (SANS), and single-molecule Förster resonance energy transfer (smFRET) measurements allow for the estimation or calculation of <Rg> for the denatured state ensemble in the aqueous solutions containing variable concentrations of urea or GdmCl [33,61,62,63,64]. In the smFRET experiments, the fluorescence energy transfer efficiency depends upon the distance between the donor and acceptor dye groups (that are covalently attached to two distant points of the chain), and such dependence can suitably be exploited to estimate the <Rg> value. Data analysis requires the use of a theoretical probability distribution function for the end-to-end distance that has to become a probability distribution for the radius of gyration of the polymer chains [65,66]. Schuler and colleagues used the relationships of the coil-to-globule transition theory of Sanchez [67], arriving at a Flory–Fisk probability distribution for Rg [68]. Different choices have been performed by the same research group [69] and by other research groups [70]. By analyzing experimental SAXS and smFRET data on the denatured state ensemble of different proteins, at room temperature, in aqueous solutions with high concentrations of urea or GdmCl, it has been found that <Rg(D-state)> ≈ 2(Å)·*N*_res_^3/5^ [68,71], as expected for chains in a good solvent.

In the case of the denatured state ensemble produced on increasing the temperature, it seems that the ν exponent is smaller and the chain expansion in water at high temperature is smaller than that recorded at room temperature in the presence of high concentrations of urea or GdmCl [72]. This confirms that water is a special solvent for globular proteins and that aqueous solutions of urea or GdmCl are different from water, because the latter chemical agents “bind” to protein surfaces, causing a marked swelling. Indeed, Best, Schuler, and colleagues were able to reproduce the trend of <Rg> versus GdmCl concentration of a small globular protein (obtained using smFRET measurements), in the assumption that Gdm^+^ ions bind to independent and identical binding sites on protein surface [73]. Therefore, the analysis of the SAXS and smFRET data is in line with the scenario that emerged from the structural information mined by us from PDB [36].

## 4. Intrinsically Disordered Proteins: Conformational Preferences in Different Contexts

Studies carried out in the last two decades have demonstrated that proteins can work without adopting unique 3D structures. Unstructured, and natively unfolded proteins, generally denoted as intrinsically disordered proteins (IDPs), are recognized as widely spread, functional, and evolutionary conserved. IDPs represent around 30% of the human proteome and are largely involved in both physiological and pathological states, including cancer and neurodegenerative disorders [74]. Interestingly, it has been recently discovered that IDPs also play an important role in condensation phenomena such as liquid–liquid phase separation that are believed to have remarkable functional implications [75].

IDPs are not able to adopt a unique 3D structure but rather populate ensembles of swollen and interconverting conformations [76]. From a molecular point of view, IDPs are characterized by the enrichment in charged and hydrophilic residues (i.e., Q, E, K, R, A, G, S), which hinder the formation of a hydrophobic core and promote extended conformations due to the good electrostatic attractions with water molecules. Swollen and extended conformations are also favored by a high content of proline, a residue that destabilizes common secondary structure elements [1,6,77]. Therefore, the amino acid sequence governs protein propensity to adopt either compact/ordered or extended/disordered conformations [78].

The classical structure-function paradigm requires a significant revisitation for IDPs. The absence of a stable structure does not correspond to the lack of functionality, rather, it opens a large spectrum of possibilities [79]. IDPs can switch between different shapes able to interact with different macromolecules that govern diverse biomolecular mechanisms and activities. Structural plasticity is key to the unique functional repertoire of IDPs. The large conformational heterogeneity and structural dynamics represent a challenge for the experimental characterization of this class of proteins. Most IDP residues are solvent-exposed, producing a large surface area that, together with the absence of strong intramolecular interactions, makes these macromolecules inherently responsive to the presence of binding partners and to the chemical environment, i.e., the presence of counterions, osmolytes, and membranes.

The peculiar structural properties of IDPs prevent the use of the techniques traditionally used for the characterization of globular proteins. From the experimental point of view, SAXS and SANS, NMR, smFRET, FTIR, dynamic light scattering (DLS), hydrogen-exchange mass spectrometry (HXMS), two-focus fluorescence correlation spectroscopy (2f-FCS), photoinduced electron transfer (PET), and circular dichroism (CD) are standard methodologies routinely employed to interrogate polypeptide structures in a solution and provide quantitative insights into the conformational heterogeneity of IDPs. Such techniques do not provide an atomic-level picture of the conformational ensemble that IDPs can adopt [33,62]. Experimental observables represent ensemble averages and do not allow a complete reconstruction of the distribution function representing the conformational ensemble [80,81]. NMR techniques could provide an atomic-level description of the conformational ensemble. Indeed, the measurement of several NMR observables, dependent on dihedral angle values (i.e., chemical shifts, scalar couplings, and residual dipolar couplings), allows for the construction of physically-based structural ensembles for IDPs [82]. Usually, a huge ensemble of disordered conformations is generated by means of Monte Carlo or MD simulations, or specialized algorithms, such as the flexible-meccano [83]; then, specific sub-ensembles for the protein of interest are selected on the basis of the restraints provided by the measured NMR observables. On the other hand, careful determination of paramagnetic relaxation enhancements (PRE) can provide information about the distance distribution function between the paramagnetic probe and the nucleus of interest [84,85,86]. The latter distance distribution, coupled with MD or Monte Carlo PRE-restrained simulations, can produce a distribution function of the radius of gyration. Such an approach has been applied to α-synuclein and protein Tau by further combining NMR data with SAXS data [87,88,89]. Interestingly, it emerged that the Rg distribution obtained via SAXS is broader than that obtained via NMR because very extended conformations do not produce detectable PRE signals (in contrast, SAXS measurements, via Kratky plot, account for the overall dimensions of the scattering molecules [90,91]). Therefore, the right choice is the integrated use of NMR, SAXS, and smFRET data, as demonstrated by the successful determination of the conformational ensemble of the disordered domain of the measles virus [92].

In this framework, in silico studies are important to provide a more detailed description of IDP structures and dynamics [61,93]. MD simulations and enhanced sampling techniques, such as parallel tempering or replica exchange incorporating experimental data to restrain the simulation or to reweigh the resulting structural ensembles, are typically used to improve IDP conformational sampling. Intense research is aimed at overcoming the lack of accuracy of current MD force-fields and defining the best practice to achieve better sampling [94,95,96].

Changes in the environmental settings are proven to play a significant role in the conformational adaptation of IDPs. These are characterized by the responsiveness to variations in external conditions, such as temperature, solution pH, presence of counterions and osmolytes, and macromolecular crowding, i.e., the effect of adding macromolecules, such as proteins, nucleic acids, and carbohydrates, to create a crowded medium, with a limited amount of free water [2]. Recent data from experiments, simulations, and theory coherently indicate that the effects of macromolecular crowding, including self-crowding, are important to mediate the compaction and the formation of local structures [97,98,99,100,101,102]. A remarkable example is given by the study of Histatin 5 under self-crowding conditions: quasi-elastic neutron scattering and full atomistic MD simulations demonstrate that the diffusion rate of Histatin 5 greatly decreases on increasing crowding [103]. Moreover, Schuler and colleagues addressed the crowding effects on four different IDPs (i.e., ProTα-N and ProTα-C, ACTR, and IN) using smFRET measurements, reporting the compaction of the proteins by increasing both the concentrations and sizes of the crowding agents [99]. The responsiveness of four other different IDPs (i.e., P53, PUMA, Ash1, and E1A) to changes in the chemical composition of the surrounding solutions has been assessed using experimental, computational, and analytical studies. Recorded changes in chain dimension proved that the amino acid sequence rules the sensitivity to the chemical environment [104]. Remarkably, smFRET measurements, analyzed by a suitable polymer theory approach, and MD simulations demonstrated that five different IDPs (i.e., MYC, MAX, MAD, MLX, and MONDOA) expand on increasing the solution ionic strength, due to Debye–Hückel charge screening [105].

### Effects of Chemical Denaturants on IDPs

Polymer physics approaches have been used to characterize the conformational ensembles populated by IDPs. Schuler and colleagues, employing smFRET measurements on increasing the GdmCl concentration, determined the Rg of two highly charged IDPs, and found a scaling law close to that valid for the denatured state ensemble of foldable proteins in high concentrations of urea or GdmCl [68]. Actually, the obtained ν exponent was larger than 3/5 (i.e., the value expected for a polymer in a good solvent), emphasizing the strong attractive interactions existing between the charged protein surfaces and the Gdm^+^ ions and water molecules. It emerged that the ν exponent value increases with the average net charge of the polypeptide chain, and decreases with the average hydrophobicity of the polypeptide chain [68]. Moreover, on decreasing the GdmCl concentration, Schuler and colleagues found that the ν exponent decreases for the two IDPs, but it remains significantly larger than 1/2 (i.e., the value expected for a theta solvent) also in water. This behavior of IDPs contrasts with that of the denatured state of foldable proteins: the ν exponent values of the latter in the denatured state are close to 1/2 in water. The authors considered this finding an indication that IDPs, possessing a high value of average net charge and a small value of average hydrophobicity, should be remnants of ancestral proteins. Indeed, the latter should be made of a limited alphabet (i.e., 10–12 amino acids), containing acid residues, polar residues, small nonpolar ones, and no aromatic residues [68,106].

The analysis of the smFRET measurements, using suitable polymer physics approaches, showed that <Rg> decreases on decreasing the urea or GdmCl concentration of the aqueous solutions for both the denatured state ensemble of foldable proteins and IDPs [58,85,107]. These results were in contrast with those obtained from the analysis of the SAXS measurements. In the latter case, <Rg> does not decrease by lowering the urea or GdmCl concentration of aqueous solutions [108,109,110]. It should be clear that: (a) the two techniques are different and need protein solutions with very different concentrations to obtain detectable signals; (b) experimental raw data, in both cases, requires careful and not trivial analysis. To address the matter, Best, Schuler, and colleagues performed a careful investigation on the action of urea and GdmCl on ACTR, a small IDP of 73 residues, through smFRET and SAXS measurements, and long MD simulations in explicit water (i.e., the TIP4P-2005 water model [111]), and explicit Kirkwood–Buff force-fields for the two denaturants [33,63]. They found that there is no conflict between the results obtained from the analysis of SAXS and smFRET measurements. In particular, in both cases: (a) the <Rg> values show an increasing trend with the concentration of both denaturants; (b) the <Rg> value at high denaturant concentration is in line with the scaling law for a polymer in a good solvent [62,63]. In several different studies, the authors addressed the denaturant-induced expansion of both unfolded and disordered states by combining several techniques. They showed that current experimental measurements, allowing a molecular-level description of the denaturant-mediated mechanism, can largely benefit from improved all-atom simulations, which can provide an atomistic perspective to solve actual discrepancies [62,66,112].

Further information on the interplay between the information obtained from the SAXS and smFRET data analyses has been provided by Pappu, Svergun, Lemke, and colleagues, who tried to address the conflicting outcomes between SAXS and smFRET by performing both measurements on ten different proteins and all-atom Monte Carlo simulations on five IDPs [113]. They found that: (a) the agreement between SAXS and smFRET exists in aqueous solutions with high denaturant concentration, and is lost on decreasing the denaturant concentration and moving toward native conditions; (b) this is not due to the presence of the two dyes in the protein samples used in smFRET measurements; (c) it is due to limitations caused by the choice of the probability distribution function necessary to analyze smFRET data. Specifically, the fluorescence energy transfer efficiency measured in smFRET depends on the end-to-end distance of the chain and this size measure has to be related to the radius of gyration. Such a connection becomes problematic when the conformational ensemble is highly heterogeneous [113,114]. Indeed, it has been shown that it is possible to have ensembles for which the values of the average end-to-end distance are markedly different, even though the values of <Rg> are almost the same. The probability of such a situation increases when the populated conformations are largely non-spherical (i.e., when the polypeptide chains are in between the fully native and fully denatured conditions). The conclusion is that SAXS and smFRET measurements provide different views that can be merged thanks to suitable MD or Monte Carlo simulations, to gain more detailed structural information on IDP conformational ensembles.

## 5. Effects of Chemical Denaturants on Amyloid-Like Aggregates

Investigations carried out in the last decades and focused on the search for the molecular basis underlying severe and widespread neurodegenerative diseases have unraveled the existence of a highly rigid and compact protein structure motif denoted as cross-β [3,115]. This type of structure is characterized by β-sheets in which the polypeptide chains of the β-strands run perpendicularly to the growth axis of the aggregates [116]. These β-sheets, which can be either parallel or antiparallel, are stabilized by the typical network of backbone H-bonds and, occasionally, by interactions, either polar or hydrophobic, made by the side chain of residues belonging to different strands [117]. These assemblies, which can be either soluble or insoluble, are further stabilized by lateral interactions made by pairs of facing β-sheets. Again, these inter-sheet interactions may be either polar or nonpolar depending on the involved residues. Importantly, these cross-β assemblies, also denoted as amyloids, based on the analogy with the aggregates detected in some neurodegenerative diseases, may occur in protein functional states [118]. The intrinsic ability of polypeptide chains to form amyloid-like assemblies has been corroborated by the discovery that this type of structure may be formed by small peptides endowed with sequences characterized by highly diversified polarities [118,119]. These have opened new avenues for biomedical and technological applications and have demonstrated the almost universal propensity of peptide chains to adopt this structural motif [120]. The wide interest in this motif has stimulated a plethora of investigations aimed at unraveling the physico-chemical basis of their unusual stability as well as their response to environmental effectors, such as chemical denaturants [121]. Such investigations have covered the role of denaturants, mainly urea and guanidinium, on different aspects of amyloid formation, stabilization, and disaggregation.

Compared with the folding/unfolding process of globular proteins, aggregation/disaggregation processes exhibited by amyloids have some important specificities. While the folding/unfolding of globular proteins is unimolecular, amyloid aggregation involves many molecules. Moreover, as highlighted above, whereas folded states of globular proteins are marginally stabilized compared to the denatured state ensembles, amyloid aggregates are characterized by extraordinary thermal and chemical stability [122]. Finally, amyloid formation strongly depends on the starting states (folded globular, unfolded, or intrinsically disordered) of the aggregating protein. The aggregation process is generally dissected in two main steps: the time interval required for the formation of the first nuclei of the aggregates (nucleation/lag phase), and an elongation phase which is characterized by the growth of aggregates [123]. This elongation phase may also take place through secondary nucleation, where initial aggregates may constitute the catalytic surface for the formation of new aggregates [124].

Chemical denaturants may operate at different stages of the amyloid formation process and may either favor or disfavor it [125]. In some cases, it has been observed a reduction of the lag phase. This is likely due to the increment of the concentration of the protein in the unfolded state which is more aggregation-prone. This was observed in the aggregation of Cu/Zn superoxide dismutase upon the addition of urea [126]. Interestingly, the increase of the aggregation rate caused by the denaturant addition is not monotone with its concentration. For example, the urea addition to β-lactoglobulin increases its aggregation rate only when used at a concentration below 5 M [127]. When urea is used above 5 M, the protein aggregation rate decreases due to the ability of the denaturant to optimally solvate unfolded conformations. Similar behavior has been observed upon the addition of guanidinium in the aggregation process of hen-egg-white lysozyme; an increase or a decrease of protein aggregation occurs at concentrations of the denaturant below and above 3 M, respectively [128].

A comprehensive analysis of the impact that urea and guanidinium have on the aggregation process of the peptide Aβ42, a natively unfolded peptide whose aggregation is believed to be heavily implicated in the insurgence and progression of Alzheimer’s disease, has been reported by Linse, Knowles, and colleagues [125]. These authors demonstrated that urea, as a non-ionic denaturant, reduces the overall aggregation rate with a stronger effect on nucleation compared to the elongation steps. On the other hand, the ionic denaturant, guanidinium, accelerates the aggregation at low concentrations and decelerates it at high concentrations. These findings have been explained by assuming that, at low concentrations, Gdm^+^ operates by screening repulsive electrostatic interactions between different molecules of Aβ42, which present an overall charge between −3 and −4 at pH 8.0, leading to an increased aggregation rate. At higher Gdm^+^ concentrations, the electrostatic repulsion is completely screened, and the denaturing effect dominates. The authors tried to mimic the effects of guanidinium by using an equimolar mixture of NaCl and urea. The effects produced by GdmCl are not fully reproduced by this mixture. This indicates that Gdm^+^ has additional effects on the peptide, likely related to its ability to interact with polar groups, as highlighted by the analysis of protein-guanidinium adducts present in the PDB [34,36], which are particularly abundant in Aβ42. This affinity may prevent self-interactions between peptide residues, favoring more extended conformations.

Regarding the ability of chemical denaturants to disaggregate formed amyloids, sometimes referred to as depolymerization, intriguing specificities have been found compared to the unfolding process of globular proteins. Although some special amyloids, typically those involved in biofilm formation, are resistant to very strong chemical denaturants [129], several investigations on the ability of common chemical denaturants to disaggregate amyloids have been reported [130,131]. While for protein unfolding a general trend for the relative strength of chemical denaturants is observed, with guanidinium being more powerful than urea, opposite trends are frequently observed for amyloid disaggregation. It has been observed a stabilizing role of Gdm^+^ for amyloid fibrils when used at moderate concentrations [132]. The limited effectiveness of Gdm^+^ to perturb amyloid structure indicates that electrostatic interactions have a reduced role in the stabilization of these assemblies compared to the impact they have in the stabilization of the native state of globular proteins [132]. It is also possible that the increased ability of Gdm^+^ compared with urea to interact with nonpolar groups (see Figure 3) has a role in this distinctive behavior [36]. Although amyloids may be reinforced by hydrophobic interactions, polar interactions, such as H-bonds, are certainly prevalent in these structures. Moreover, in contrast to globular proteins that are almost universally characterized by a hydrophobic core, amyloids can be formed by peptides composed only of polar residues (i.e., polyglutamine) [133,134].

## 6. Conclusions

The leitmotif of the present review is the analysis of the impact that chemical denaturants have on protein structure in all of its manifestations. In addition to the widely studied globular proteins, we have also explored available literature on more recently emerged protein structural states, such as IDPs and amyloid-like assemblies, whose functional and mis-functional relevance is by now well established. Literature data have been illustrated with particular attention to the proposed molecular and physical mechanisms underlying the action(s) of chemical denaturants. A comparative analysis of denaturant-induced effects emphasizes the occurrence of similarities and differences in the different classes of protein structures. The different forces that stabilize these distinct structural states generate intriguing effects. Even the ranking of the relative strength of the most common denaturants, with the guanidinium stronger than urea, which is well-established and generally conserved for globular proteins, is not fully respected for amyloid-like assemblies. For the latter, denaturant effects are more intricate, as the formation and disaggregation of amyloid-like assemblies involve multiple and multi-molecular steps.

Data and the potential mechanism of the denaturants were analyzed and illustrated using both a polymer physics and molecular perspective. The polymer physics approach indicates the effectiveness of the “poor” and “good” solvent concepts quantified in terms of the ν exponent of the well-established relationship <Rg> ∝ *N*^ν^, which links the average radius of gyration to the number of protein residues. In this perspective, water, which is a poor solvent for globular proteins, becomes a good solvent upon the addition of chemical denaturants (protein unfolding) or is a good solvent for IDPs, whose sequences are characterized by an unusual abundance of charged residues. Recent investigations have shown that the addition of chemical denaturants to IDPs causes a further increase in the ν exponent, demonstrating that these compounds have a precise impact on the conformational ensemble of these proteins [68].

From the molecular point of view, recent extensive analyses of the Protein Data Bank have shown the strong tendency of chemical denaturing agents to interact with protein moieties endowed with rather different physico-chemical properties [34,35,36]. Particularly evident is the propensity of the strongest chemical denaturants, such as guanidinium and thiocyanate, to interact with nonpolar groups. The promiscuity of these compounds allows for the exposure of normally poorly solvated groups that, in turn, favors the opening of folded structures and the stability of extended and swollen conformations. In this way, these compounds mediate the interactions between protein moieties and water, rendering the latter a good solvent.

Even though the illustrated findings indicate that, in recent years, significant progress has been made, some questions are still not completely answered [62,95,108,112,113]. In particular, the response of IDPs to environmental perturbations is of great importance considering their labile structural preferences and their functional importance. Experimental difficulties in obtaining atomic-level information on these proteins make our understanding still limited. In this scenario, computational and theoretical methods, which provided remarkable contributions in the characterization of globular proteins, represent a valuable tool to study the structural features of IDPs. Significant advances in the development of atomistic force-fields, ad-hoc for these systems, have been made [63,93,96,112,135,136]. Additional efforts are necessary to further improve computational approaches that represent the only resource to gain an atomic-level picture of these puzzling proteins.

## Figures and Tables

**Table 1:** Experimental values, at 25 °C and 1 atm, of the density and water molar concentration for pure water and aqueous 2, 4, and 6 M urea solutions, and 2, 4, and 6 M GdmCl solutions [20,21]; values of the volume packing density for all these liquid solutions, calculated using the following effective hard sphere diameters: σ(H_2_O) = 2.80 Å, σ(urea) = 4.64 Å, σ(Gdm^+^) = 4.70 Å, and σ(Cl^−^) = 3.62 Å [26,27]; experimental values of surface tension at 20 °C [22,23].

	ρ g L−1	[H_2_O] M	ξ3	γ Dyne cm−1
H_2_O	997	55.3	0.383	72.8
2 M urea	1029	50.4	0.413	72.9
4 M urea	1059	45.4	0.442	73.3
6 M urea	1088	40.4	0.471	74.4
2 M GdmCl	1047	47.5	0.424	74.0
4 M GdmCl	1095	39.6	0.465	74.8
6 M GdmCl	1140	31.5	0.504	75.0

## Data Availability

Data sharing not applicable.

## References

[1] Tompa P. (2003). Intrinsically Unstructured Proteins Evolve by Repeat Expansion. BioEssays.

[2] Uversky V.N. (2009). Intrinsically Disordered Proteins and Their Environment: Effects of Strong Denaturants, Temperature, PH, Counter Ions, Membranes, Binding Partners, Osmolytes, and Macromolecular Crowding. Protein J..

[3] Sawaya M.R., Hughes M.P., Rodriguez J.A., Riek R., Eisenberg D.S. (2021). The Expanding Amyloid Family: Structure, Stability, Function, and Pathogenesis. Cell.

[4] Tompa P. (2002). Intrinsically Unstructured Proteins. Trends Biochem. Sci..

[5] Dunker A.K., Babu M.M., Barbar E., Blackledge M., Bondos S.E., Dosztányi Z., Dyson H.J., Forman-Kay J., Fuxreiter M., Gsponer J. (2013). What’s in a Name? Why These Proteins Are Intrinsically Disordered: Why These Proteins Are Intrinsically Disordered. Intrinsically Disord. Proteins.

[6] Quaglia F., Mészáros B., Salladini E., Hatos A., Pancsa R., Chemes L.B., Pajkos M., Lazar T., Peña-Díaz S., Santos J. (2022). DisProt in 2022: Improved Quality and Accessibility of Protein Intrinsic Disorder Annotation. Nucleic Acids Res..

[7] Otzen D., Riek R. (2019). Functional Amyloids. Cold Spring Harb. Perspect. Biol..

[8] Bhatia S., Udgaonkar J.B. (2022). Heterogeneity in Protein Folding and Unfolding Reactions. Chem. Rev..

[9] Dill K.A., MacCallum J.L. (2012). The Protein-Folding Problem, 50 Years On. Science.

[10] Gianni S., Dogan J., Jemth P. (2016). Coupled Binding and Folding of Intrinsically Disordered Proteins: What Can We Learn from Kinetics?. Curr. Opin. Struct. Biol..

[11] Chiti F., Dobson C.M. (2017). Protein Misfolding, Amyloid Formation, and Human Disease: A Summary of Progress Over the Last Decade. Annu. Rev. Biochem..

[12] Lazar T., Martínez-Pérez E., Quaglia F., Hatos A., Chemes L.B., Iserte J.A., Méndez N.A., Garrone N.A., Saldaño T.E., Marchetti J. (2021). PED in 2021: A Major Update of the Protein Ensemble Database for Intrinsically Disordered Proteins. Nucleic Acids Res..

[13] Zheng Z., Zhang M., Wang Y., Ma R., Guo C., Feng L., Wu J., Yao H., Lin D. (2018). Structural Basis for the Complete Resistance of the Human Prion Protein Mutant G127V to Prion Disease. Sci. Rep..

[14] Makhatadze G.I., Privalov P.L. (1995). Energetics of Protein Structure. Advances in Protein Chemistry.

[15] Robertson A.D., Murphy K.P. (1997). Protein Structure and the Energetics of Protein Stability. Chem. Rev..

[16] Rees D.C., Robertson A.D. (2001). Some Thermodynamic Implications for the Thermostability of Proteins. Protein Sci..

[17] Sawle L., Ghosh K. (2011). How Do Thermophilic Proteins and Proteomes Withstand High Temperature?. Biophys. J..

[18] Pauling L. (1960). The Nature of the Chemical Bond.

[19] Shortle D. (1996). The Denatured State (the Other Half of the Folding Equation) and Its Role in Protein Stability. FASEB J..

[20] Kawahara K., Tanford C. (1966). Viscosity and Density of Aqueous Solutions of Urea and Guanidine Hydrochloride. J. Biol. Chem..

[21] Kumar A. (2001). Aqueous Guanidinium Salts: Part I. Densities, Ultrasonic Velocities, and Apparent Molar Properties. J. Solut. Chem..

[22] Halonen S., Kangas T., Haataja M., Lassi U. (2017). Urea-Water-Solution Properties: Density, Viscosity, and Surface Tension in an Under-Saturated Solution. Emiss. Control Sci. Technol..

[23] Breslow R., Guo T. (1990). Surface Tension Measurements Show That Chaotropic Salting-in Denaturants Are Not Just Water-Structure Breakers. Proc. Natl. Acad. Sci. USA.

[24] Soper A.K., Castner E.W., Luzar A. (2003). Impact of Urea on Water Structure: A Clue to Its Properties as a Denaturant?. Biophys. Chem..

[25] Mason P.E., Neilson G.W., Enderby J.E., Saboungi M.-L., Dempsey C.E., MacKerell A.D., Brady J.W. (2004). The Structure of Aqueous Guanidinium Chloride Solutions. J. Am. Chem. Soc..

[26] Graziano G. (2011). Contrasting the Denaturing Effect of Guanidinium Chloride with the Stabilizing Effect of Guanidinium Sulfate. Phys. Chem. Chem. Phys..

[27] Graziano G. (2011). How Does Trimethylamine N-Oxide Counteract the Denaturing Activity of Urea?. Phys. Chem. Chem. Phys..

[28] Graziano G. (2001). On the Solubility of Aliphatic Hydrocarbons in 7 M Aqueous Urea. J. Phys. Chem. B.

[29] Trzesniak D., van der Vegt N.F.A., van Gunsteren W.F. (2004). Computer Simulation Studies on the Solvation of Aliphatic Hydrocarbons in 6.9 M Aqueous Urea Solution. Phys. Chem. Chem. Phys..

[30] Weerasinghe S., Smith P.E. (2003). A Kirkwood−Buff Derived Force Field for Mixtures of Urea and Water. J. Phys. Chem. B.

[31] Weerasinghe S., Smith P.E. (2004). A Kirkwood-Buff Derived Force Field for the Simulation of Aqueous Guanidinium Chloride Solutions. J. Chem. Phys..

[32] Zangi R., Zhou R., Berne B.J. (2009). Urea’s Action on Hydrophobic Interactions. J. Am. Chem. Soc..

[33] Zheng W., Borgia A., Borgia M.B., Schuler B., Best R.B. (2015). Empirical Optimization of Interactions between Proteins and Chemical Denaturants in Molecular Simulations. J. Chem. Theory Comput..

[34] Cozzolino S., Balasco N., Vigorita M., Ruggiero A., Smaldone G., Del Vecchio P., Vitagliano L., Graziano G. (2020). Guanidinium Binding to Proteins: The Intriguing Effects on the D1 and D2 Domains of Thermotoga Maritima Arginine Binding Protein and a Comprehensive Analysis of the Protein Data Bank. Int. J. Biol. Macromol..

[35] Paladino A., Balasco N., Graziano G., Vitagliano L. (2022). A Protein Data Bank Survey of Multimodal Binding of Thiocyanate to Proteins: Evidence for Thiocyanate Promiscuity. Int. J. Biol. Macromol..

[36] Paladino A., Balasco N., Vitagliano L., Graziano G. (2022). A Structure-Based Mechanism for the Denaturing Action of Urea, Guanidinium Ion and Thiocyanate Ion. Biology.

[37] Makhatadze G.I., Privalov P.L. (1992). Protein Interactions with Urea and Guanidinium Chloride. J. Mol. Biol..

[38] Schellman J.A. (1994). The Thermodynamics of Solvent Exchange. Biopolymers.

[39] Schellman J.A. (2003). Protein Stability in Mixed Solvents: A Balance of Contact Interaction and Excluded Volume. Biophys. J..

[40] Rembert K.B., Paterová J., Heyda J., Hilty C., Jungwirth P., Cremer P.S. (2012). Molecular Mechanisms of Ion-Specific Effects on Proteins. J. Am. Chem. Soc..

[41] Courtenay E.S., Capp M.W., Record M.T. (2009). Thermodynamics of Interactions of Urea and Guanidinium Salts with Protein Surface: Relationship between Solute Effects on Protein Processes and Changes in Water-Accessible Surface Area. Protein Sci..

[42] Record M.T., Guinn E., Pegram L., Capp M. (2013). Introductory Lecture: Interpreting and Predicting Hofmeister Salt Ion and Solute Effects on Biopolymer and Model Processes Using the Solute Partitioning Model. Faraday Discuss.

[43] Pegram L.M., Record M.T. (2008). Thermodynamic Origin of Hofmeister Ion Effects. J. Phys. Chem. B.

[44] Van Der Spoel D., Lindahl E., Hess B., Groenhof G., Mark A.E., Berendsen H.J.C. (2005). GROMACS: Fast, Flexible, and Free. J. Comput. Chem..

[45] Lindorff-Larsen K., Piana S., Palmo K., Maragakis P., Klepeis J.L., Dror R.O., Shaw D.E. (2010). Improved Side-Chain Torsion Potentials for the Amber Ff99SB Protein Force Field: Improved Protein Side-Chain Potentials. Proteins Struct. Funct. Bioinform..

[46] Jorgensen W.L. (1982). Monte Carlo Simulation of *n*-butane in Water. Conformational Evidence for the Hydrophobic Effect. J. Chem. Phys..

[47] O’Brien E.P., Dima R.I., Brooks B., Thirumalai D. (2007). Interactions between Hydrophobic and Ionic Solutes in Aqueous Guanidinium Chloride and Urea Solutions: Lessons for Protein Denaturation Mechanism. J. Am. Chem. Soc..

[48] O’Brien E.P., Ziv G., Haran G., Brooks B.R., Thirumalai D. (2008). Effects of Denaturants and Osmolytes on Proteins Are Accurately Predicted by the Molecular Transfer Model. Proc. Natl. Acad. Sci. USA.

[49] Liu Z., Reddy G., Thirumalai D. (2012). Theory of the Molecular Transfer Model for Proteins with Applications to the Folding of the Src-SH3 Domain. J. Phys. Chem. B.

[50] Liu Z., Reddy G., Thirumalai D. (2016). Folding PDZ2 Domain Using the Molecular Transfer Model. J. Phys. Chem. B.

[51] Klimov D.K., Thirumalai D. (2000). Mechanisms and Kinetics of β-Hairpin Formation. Proc. Natl. Acad. Sci. USA.

[52] Tanford C. (1968). Protein Denaturation. Advances in Protein Chemistry.

[53] Flory P.J. (1953). Principles of Polymer Chemistry.

[54] Chan H.S., Dill K.A. (1991). Polymer Principles in Protein Structure and Stability. Annu. Rev. Biophys. Biophys. Chem..

[55] Clark P.L., Plaxco K.W., Sosnick T.R. (2020). Water as a Good Solvent for Unfolded Proteins: Folding and Collapse Are Fundamentally Different. J. Mol. Biol..

[56] Graziano G. (2014). On the Mechanism of Cold Denaturation. Phys. Chem. Chem. Phys..

[57] Merlino A., Pontillo N., Graziano G. (2017). A Driving Force for Polypeptide and Protein Collapse. Phys. Chem. Chem. Phys..

[58] Graziano G. (2020). Is Water a Good Solvent for the Denatured State of Globular Proteins?. Chem. Phys. Lett..

[59] Dima R.I., Thirumalai D. (2004). Asymmetry in the Shapes of Folded and Denatured States of Proteins. J. Phys. Chem. B.

[60] Holehouse A.S., Pappu R.V. (2018). Collapse Transitions of Proteins and the Interplay Among Backbone, Sidechain, and Solvent Interactions. Annu. Rev. Biophys..

[61] Best R.B. (2020). Emerging Consensus on the Collapse of Unfolded and Intrinsically Disordered Proteins in Water. Curr. Opin. Struct. Biol..

[62] Borgia A., Zheng W., Buholzer K., Borgia M.B., Schüler A., Hofmann H., Soranno A., Nettels D., Gast K., Grishaev A. (2016). Consistent View of Polypeptide Chain Expansion in Chemical Denaturants from Multiple Experimental Methods. J. Am. Chem. Soc..

[63] Zheng W., Borgia A., Buholzer K., Grishaev A., Schuler B., Best R.B. (2016). Probing the Action of Chemical Denaturant on an Intrinsically Disordered Protein by Simulation and Experiment. J. Am. Chem. Soc..

[64] Yu L., Brüschweiler R. (2022). Quantitative Prediction of Ensemble Dynamics, Shapes and Contact Propensities of Intrinsically Disordered Proteins. PLoS Comput. Biol..

[65] Merchant K.A., Best R.B., Louis J.M., Gopich I.V., Eaton W.A. (2007). Characterizing the Unfolded States of Proteins Using Single-Molecule FRET Spectroscopy and Molecular Simulations. Proc. Natl. Acad. Sci. USA.

[66] Zheng W., Zerze G.H., Borgia A., Mittal J., Schuler B., Best R.B. (2018). Inferring Properties of Disordered Chains from FRET Transfer Efficiencies. J. Chem. Phys..

[67] Sanchez I.C. (1979). Phase Transition Behavior of the Isolated Polymer Chain. Macromolecules.

[68] Hofmann H., Soranno A., Borgia A., Gast K., Nettels D., Schuler B. (2012). Polymer Scaling Laws of Unfolded and Intrinsically Disordered Proteins Quantified with Single-Molecule Spectroscopy. Proc. Natl. Acad. Sci. USA.

[69] Aznauryan M., Delgado L., Soranno A., Nettels D., Huang J., Labhardt A.M., Grzesiek S., Schuler B. (2004). Comprehensive Structural and Dynamical View of an Unfolded Protein from the Combination of Single-Molecule FRET, NMR, and SAXS. Proc. Natl. Acad. Sci. USA.

[70] O’Brien E.P., Morrison G., Brooks B.R., Thirumalai D. (2009). How Accurate Are Polymer Models in the Analysis of Förster Resonance Energy Transfer Experiments on Proteins?. J. Chem. Phys..

[71] Kohn J.E., Millett I.S., Jacob J., Zagrovic B., Dillon T.M., Cingel N., Dothager R.S., Seifert S., Thiyagarajan P., Sosnick T.R. (2004). Random-Coil Behavior and the Dimensions of Chemically Unfolded Proteins. Proc. Natl. Acad. Sci. USA.

[72] Narayan A., Bhattacharjee K., Naganathan A.N. (2019). Thermally versus Chemically Denatured Protein States. Biochemistry.

[73] Nettels D., Müller-Späth S., Küster F., Hofmann H., Haenni D., Rüegger S., Reymond L., Hoffmann A., Kubelka J., Heinz B. (2009). Single-Molecule Spectroscopy of the Temperature-Induced Collapse of Unfolded Proteins. Proc. Natl. Acad. Sci. USA.

[74] Tompa P. (2012). Intrinsically Disordered Proteins: A 10-Year Recap. Trends Biochem. Sci..

[75] Turoverov K.K., Kuznetsova I.M., Fonin A.V., Darling A.L., Zaslavsky B.Y., Uversky V.N. (2019). Stochasticity of Biological Soft Matter: Emerging Concepts in Intrinsically Disordered Proteins and Biological Phase Separation. Trends Biochem. Sci..

[76] Uversky V.N. (2019). Intrinsically Disordered Proteins and Their “Mysterious” (Meta)Physics. Front. Phys..

[77] Forman-Kay J.D., Mittag T. (2013). From Sequence and Forces to Structure, Function, and Evolution of Intrinsically Disordered Proteins. Structure.

[78] Das R.K., Ruff K.M., Pappu R.V. (2015). Relating Sequence Encoded Information to Form and Function of Intrinsically Disordered Proteins. Curr. Opin. Struct. Biol..

[79] Kulkarni P., Bhattacharya S., Achuthan S., Behal A., Jolly M.K., Kotnala S., Mohanty A., Rangarajan G., Salgia R., Uversky V. (2022). Intrinsically Disordered Proteins: Critical Components of the Wetware. Chem. Rev..

[80] Gomes G.-N.W., Krzeminski M., Namini A., Martin E.W., Mittag T., Head-Gordon T., Forman-Kay J.D., Gradinaru C.C. (2020). Conformational Ensembles of an Intrinsically Disordered Protein Consistent with NMR, SAXS, and Single-Molecule FRET. J. Am. Chem. Soc..

[81] Shrestha U.R., Smith J.C., Petridis L. (2021). Full Structural Ensembles of Intrinsically Disordered Proteins from Unbiased Molecular Dynamics Simulations. Commun. Biol..

[82] Milles S., Salvi N., Blackledge M., Jensen M.R. (2018). Characterization of Intrinsically Disordered Proteins and Their Dynamic Complexes: From in Vitro to Cell-like Environments. Prog. Nucl. Magn. Reson. Spectrosc..

[83] Ozenne V., Bauer F., Salmon L., Huang J., Jensen M.R., Segard S., Bernadó P., Charavay C., Blackledge M. (2012). *Flexible-Meccano:* A Tool for the Generation of Explicit Ensemble Descriptions of Intrinsically Disordered Proteins and Their Associated Experimental Observables. Bioinformatics.

[84] Allison J.R., Varnai P., Dobson C.M., Vendruscolo M. (2009). Determination of the Free Energy Landscape of α-Synuclein Using Spin Label Nuclear Magnetic Resonance Measurements. J. Am. Chem. Soc..

[85] Ganguly D., Chen J. (2009). Structural Interpretation of Paramagnetic Relaxation Enhancement-Derived Distances for Disordered Protein States. J. Mol. Biol..

[86] Silvestre-Ryan J., Bertoncini C.W., Fenwick R.B., Esteban-Martin S., Salvatella X. (2013). Average Conformations Determined from PRE Data Provide High-Resolution Maps of Transient Tertiary Interactions in Disordered Proteins. Biophys. J..

[87] Schwalbe M., Ozenne V., Bibow S., Jaremko M., Jaremko L., Gajda M., Jensen M.R., Biernat J., Becker S., Mandelkow E. (2014). Predictive Atomic Resolution Descriptions of Intrinsically Disordered HTau40 and α-Synuclein in Solution from NMR and Small Angle Scattering. Structure.

[88] Cho M.-K., Nodet G., Kim H.-Y., Jensen M.R., Bernado P., Fernandez C.O., Becker S., Blackledge M., Zweckstetter M. (2009). Structural Characterization of α-Synuclein in an Aggregation Prone State: α-Synuclein in an Aggregation-Prone State. Protein Sci..

[89] Bibow S., Ozenne V., Biernat J., Blackledge M., Mandelkow E., Zweckstetter M. (2011). Structural Impact of Proline-Directed Pseudophosphorylation at AT8, AT100, and PHF1 Epitopes on 441-Residue Tau. J. Am. Chem. Soc..

[90] Sibille N., Bernadó P. (2012). Structural Characterization of Intrinsically Disordered Proteins by the Combined Use of NMR and SAXS. Biochem. Soc. Trans..

[91] Cordeiro T.N., Herranz-Trillo F., Urbanek A., Estaña A., Cortés J., Sibille N., Bernadó P. (2017). Small-Angle Scattering Studies of Intrinsically Disordered Proteins and Their Complexes. Curr. Opin. Struct. Biol..

[92] Naudi-Fabra S., Tengo M., Jensen M.R., Blackledge M., Milles S. (2021). Quantitative Description of Intrinsically Disordered Proteins Using Single-Molecule FRET, NMR, and SAXS. J. Am. Chem. Soc..

[93] Shea J.-E., Best R.B., Mittal J. (2021). Physics-Based Computational and Theoretical Approaches to Intrinsically Disordered Proteins. Curr. Opin. Struct. Biol..

[94] Mercadante D., Wagner J.A., Aramburu I.V., Lemke E.A., Gräter F. (2017). Sampling Long- versus Short-Range Interactions Defines the Ability of Force Fields to Reproduce the Dynamics of Intrinsically Disordered Proteins. J. Chem. Theory Comput..

[95] Robustelli P., Piana S., Shaw D.E. (2018). Developing a Molecular Dynamics Force Field for Both Folded and Disordered Protein States. Proc. Natl. Acad. Sci. USA.

[96] Pesce F., Lindorff-Larsen K. (2022). Refining Conformational Ensembles of Flexible Proteins against Small-Angle X-ray Scattering Data. Biophys. J..

[97] Wang Y., Benton L.A., Singh V., Pielak G.J. (2012). Disordered Protein Diffusion under Crowded Conditions. J. Phys. Chem. Lett..

[98] Szasz C., Alexa A., Toth K., Rakacs M., Langowski J., Tompa P. (2011). Protein Disorder Prevails under Crowded Conditions. Biochemistry.

[99] Soranno A., Koenig I., Borgia M.B., Hofmann H., Zosel F., Nettels D., Schuler B. (2014). Single-Molecule Spectroscopy Reveals Polymer Effects of Disordered Proteins in Crowded Environments. Proc. Natl. Acad. Sci. USA.

[100] Zosel F., Soranno A., Buholzer K.J., Nettels D., Schuler B. (2020). Depletion Interactions Modulate the Binding between Disordered Proteins in Crowded Environments. Proc. Natl. Acad. Sci. USA.

[101] Shillcock J.C., Thomas D.B., Ipsen J.H., Brown A.D. (2023). Macromolecular Crowding Is Surprisingly Unable to Deform the Structure of a Model Biomolecular Condensate. Biology.

[102] Speer S.L., Stewart C.J., Sapir L., Harries D., Pielak G.J. (2022). Macromolecular Crowding Is More than Hard-Core Repulsions. Annu. Rev. Biophys..

[103] Fagerberg E., Lenton S., Nylander T., Seydel T., Skepö M. (2022). Self-Diffusive Properties of the Intrinsically Disordered Protein Histatin 5 and the Impact of Crowding Thereon: A Combined Neutron Spectroscopy and Molecular Dynamics Simulation Study. J. Phys. Chem. B.

[104] Moses D., Yu F., Ginell G.M., Shamoon N.M., Koenig P.S., Holehouse A.S., Sukenik S. (2020). Revealing the Hidden Sensitivity of Intrinsically Disordered Proteins to Their Chemical Environment. J. Phys. Chem. Lett..

[105] Vancraenenbroeck R., Harel Y.S., Zheng W., Hofmann H. (2019). Polymer Effects Modulate Binding Affinities in Disordered Proteins. Proc. Natl. Acad. Sci. USA.

[106] Longo L.M., Blaber M. (2014). Prebiotic Protein Design Supports a Halophile Origin of Foldable Proteins. Front. Microbiol..

[107] Reddy G., Thirumalai D. (2017). Collapse Precedes Folding in Denaturant-Dependent Assembly of Ubiquitin. J. Phys. Chem. B.

[108] Riback J.A., Bowman M.A., Zmyslowski A.M., Knoverek C.R., Jumper J.M., Hinshaw J.R., Kaye E.B., Freed K.F., Clark P.L., Sosnick T.R. (2017). Innovative Scattering Analysis Shows That Hydrophobic Disordered Proteins Are Expanded in Water. Science.

[109] Yoo T.Y., Meisburger S.P., Hinshaw J., Pollack L., Haran G., Sosnick T.R., Plaxco K. (2012). Small-Angle X-Ray Scattering and Single-Molecule FRET Spectroscopy Produce Highly Divergent Views of the Low-Denaturant Unfolded State. J. Mol. Biol..

[110] Watkins H.M., Simon A.J., Sosnick T.R., Lipman E.A., Hjelm R.P., Plaxco K.W. (2015). Random Coil Negative Control Reproduces the Discrepancy between Scattering and FRET Measurements of Denatured Protein Dimensions. Proc. Natl. Acad. Sci. USA.

[111] Abascal J.L.F., Vega C. (2005). A General Purpose Model for the Condensed Phases of Water: TIP4P/2005. J. Chem. Phys..

[112] Best R.B. (2017). Computational and Theoretical Advances in Studies of Intrinsically Disordered Proteins. Curr. Opin. Struct. Biol..

[113] Fuertes G., Banterle N., Ruff K.M., Chowdhury A., Mercadante D., Koehler C., Kachala M., Estrada Girona G., Milles S., Mishra A. (2017). Decoupling of Size and Shape Fluctuations in Heteropolymeric Sequences Reconciles Discrepancies in SAXS vs. FRET Measurements. Proc. Natl. Acad. Sci. USA.

[114] Song J., Gomes G.-N., Gradinaru C.C., Chan H.S. (2015). An Adequate Account of Excluded Volume Is Necessary To Infer Compactness and Asphericity of Disordered Proteins by Förster Resonance Energy Transfer. J. Phys. Chem. B.

[115] Ke P.C., Zhou R., Serpell L.C., Riek R., Knowles T.P.J., Lashuel H.A., Gazit E., Hamley I.W., Davis T.P., Fändrich M. (2020). Half a Century of Amyloids: Past, Present and Future. Chem. Soc. Rev..

[116] Jahn T.R., Makin O.S., Morris K.L., Marshall K.E., Tian P., Sikorski P., Serpell L.C. (2010). The Common Architecture of Cross-β Amyloid. J. Mol. Biol..

[117] Taylor A.I.P., Staniforth R.A. (2022). General Principles Underpinning Amyloid Structure. Front. Neurosci..

[118] Reches M., Gazit E. (2003). Casting Metal Nanowires Within Discrete Self-Assembled Peptide Nanotubes. Science.

[119] Balasco N., Diaferia C., Morelli G., Vitagliano L., Accardo A. (2021). Amyloid-Like Aggregation in Diseases and Biomaterials: Osmosis of Structural Information. Front. Bioeng. Biotechnol..

[120] Amit M., Yuran S., Gazit E., Reches M., Ashkenasy N. (2018). Tailor-Made Functional Peptide Self-Assembling Nanostructures. Adv. Mater..

[121] Buell A.K., Dobson C.M., Knowles T.P.J. (2014). The Physical Chemistry of the Amyloid Phenomenon: Thermodynamics and Kinetics of Filamentous Protein Aggregation. Essays Biochem..

[122] Baldwin A.J., Knowles T.P.J., Tartaglia G.G., Fitzpatrick A.W., Devlin G.L., Shammas S.L., Waudby C.A., Mossuto M.F., Meehan S., Gras S.L. (2011). Metastability of Native Proteins and the Phenomenon of Amyloid Formation. J. Am. Chem. Soc..

[123] Michaels T.C.T., Šarić A., Habchi J., Chia S., Meisl G., Vendruscolo M., Dobson C.M., Knowles T.P.J. (2018). Chemical Kinetics for Bridging Molecular Mechanisms and Macroscopic Measurements of Amyloid Fibril Formation. Annu. Rev. Phys. Chem..

[124] Arosio P., Knowles T.P.J., Linse S. (2015). On the Lag Phase in Amyloid Fibril Formation. Phys. Chem. Chem. Phys..

[125] Weiffert T., Meisl G., Curk S., Cukalevski R., Šarić A., Knowles T.P.J., Linse S. (2022). Influence of Denaturants on Amyloid Β42 Aggregation Kinetics. Front. Neurosci..

[126] Khan M.A.I., Respondek M., Kjellström S., Deep S., Linse S., Akke M. (2017). Cu/Zn Superoxide Dismutase Forms Amyloid Fibrils under Near-Physiological Quiescent Conditions: The Roles of Disulfide Bonds and Effects of Denaturant. ACS Chem. Neurosci..

[127] Hamada D., Dobson C.M. (2009). A Kinetic Study of β-Lactoglobulin Amyloid Fibril Formation Promoted by Urea. Protein Sci..

[128] Vernaglia B.A., Huang J., Clark E.D. (2004). Guanidine Hydrochloride Can Induce Amyloid Fibril Formation from Hen Egg-White Lysozyme. Biomacromolecules.

[129] Dueholm M.S., Petersen S.V., Sønderkaer M., Larsen P., Christiansen G., Hein K.L., Enghild J.J., Nielsen J.L., Nielsen K.L., Nielsen P.H. (2010). Functional Amyloid in Pseudomonas: Functional Amyloid in Pseudomonas. Mol. Microbiol..

[130] Buell A.K. (2022). Stability Matters, Too–the Thermodynamics of Amyloid Fibril Formation. Chem. Sci..

[131] Sulatsky M.I., Sulatskaya A.I., Stepanenko O.V., Povarova O.I., Kuznetsova I.M., Turoverov K.K. (2020). Denaturant Effect on Amyloid Fibrils: Declasterization, Depolymerization, Denaturation and Reassembly. Int. J. Biol. Macromol..

[132] Vettore N., Buell A.K. (2019). Thermodynamics of Amyloid Fibril Formation from Chemical Depolymerization. Phys. Chem. Chem. Phys..

[133] Chen S., Berthelier V., Hamilton J.B., O’Nuallai B., Wetzel R. (2002). Amyloid-like Features of Polyglutamine Aggregates and Their Assembly Kinetics. Biochemistry.

[134] Sawaya M.R., Sambashivan S., Nelson R., Ivanova M.I., Sievers S.A., Apostol M.I., Thompson M.J., Balbirnie M., Wiltzius J.J.W., McFarlane H.T. (2007). Atomic Structures of Amyloid Cross-β Spines Reveal Varied Steric Zippers. Nature.

[135] Pesce F., Newcombe E.A., Seiffert P., Tranchant E.E., Olsen J.G., Grace C.R., Kragelund B.B., Lindorff-Larsen K. (2023). Assessment of Models for Calculating the Hydrodynamic Radius of Intrinsically Disordered Proteins. Biophys. J..

[136] Thomasen F.E., Pesce F., Roesgaard M.A., Tesei G., Lindorff-Larsen K. (2022). Improving Martini 3 for Disordered and Multidomain Proteins. J. Chem. Theory Comput..

